# Clinical relevance of single nucleotide polymorphisms within the entire *NLRP3 *gene in patients with major blunt trauma

**DOI:** 10.1186/cc10564

**Published:** 2011-11-23

**Authors:** An-Qiang Zhang, Ling Zeng, Wei Gu, Lian-Yang Zhang, Jian Zhou, Dong-po Jiang, Ding-Yuan Du, Ping Hu, Ce Yang, Jun Yan, Hai-Yan Wang, Jian-Xin Jiang

**Affiliations:** 1 State Key Laboratory of Trauma, Burns and Combined Injury, Institute of Surgery Research, Daping Hospital, Third Military Medical University, Changjiang Road 10, Yuzhong District, Chongqing, 400042, People's Republic of China; 2 Department of Traumatic Surgery, Daping Hospital, Third Military Medical University, Changjiang Road 10, Yuzhong District, Chongqing, 400042, People's Republic of China; 3 Chongqing Emergency Medical Center, Jiankang Road, Yuzhong District, Chongqing, 400042, People's Republic of China

## Abstract

**Introduction:**

The nucleotide-binding oligomerization domain-like receptor (NLR) family has been recognized as comprising intracellular pattern recognition receptors in which NLRP3 (NLR family, pyrin domain containing 3) plays an important role in the initiation of host immune inflammatory responses. The genetic variants have been recognized to be critical determinants of interindividual differences in both inflammatory responses and clinical outcomes in critical illness. However, little is known about the clinical relevance of *NLRP3 *gene polymorphisms in critical illness.

**Methods:**

A total of 718 patients with major blunt trauma were included in this study. Six tag SNPs (tSNPs) were selected from the entire *NLRP3 *gene through construction of haplotype bins, and they were genotyped using a pyrosequencing method. They were analyzed in relation to sepsis morbidity rate, multiple organ dysfunction (MOD) scores and IL-1β production. Moreover, the functionality of the rs2027432 polymorphism was assessed by the observation of its effect on transcriptional activities.

**Results:**

Among the six tSNPs genotyped in this study, two of them (rs2027432 and rs12048215) were significantly associated with sepsis morbidity rate and MOD scores. A significant association was also observed between these two polymorphisms and IL-1β production by peripheral leukocytes in response to *ex vivo *lipopolysaccharide stimulation. However, no combined effects were found between these two polymorphisms. In addition, the rs2027432 polymorphism could significantly enhance the promoter activities of the *NLRP3 *gene.

**Conclusions:**

rs2027432 and rs12048215 polymorphisms might be used as relevant risk estimates for the development of sepsis and MOD syndrome in patients with major trauma, in which rs2027432 might be a functional SNP.

## Introduction

Trauma is a major and costly public health problem worldwide, ranking as the third-leading cause of death. With great improvements in emergency care systems, the majority of severe trauma patients, even if they survive injuries, have complications after admission, leading to in-hospital death of trauma patients. Sepsis and multiple organ dysfunction syndrome (MODS) have been shown to be common and severe complications in trauma patients [1]. Therefore, preventing sepsis and MODS is crucial in the treatment of patients who survive major trauma. It has been demonstrated that inappropriate immune inflammatory response contributes to the development of sepsis and MODS in major trauma patients [2]. Increasing evidence suggests that genetic variants, particularly SNPs, are critical determinants of interindividual differences with regard to both inflammatory responses and clinical outcomes in trauma patients [3,4]. Delineating the variation in genes and associated differences in response to trauma might contribute to the development of new, genetically tailored diagnostic and therapeutic interventions that will improve outcomes in patients with major trauma.

The nucleotide-binding oligomerization domain-like receptor (NLR) family has been recognized as comprising intracellular pattern recognition receptors, which play important roles in the initiation of host immune inflammatory responses [5]. In humans, more than 20 NLRs have been identified, among which NLR family, pyrin domain containing 3 (NLRP3), is the one most often described. NLRP3 is expressed predominantly in peripheral blood leukocytes [6]. In response to invading pathogens, NLRP3 rapidly forms a cytoplasmic complex with the adapter protein apoptosis-associated specklike protein and the effector cysteine protease caspase 1, that is, the NLRP3 inflammasome, which has been shown to play a central role in the regulation of the maturation and secretion of the proinflammatory cytokines IL-1β and IL-18 [7,8]. The expression of NLRP3 has been shown to be significantly upregulated in mouse macrophages [9] and human neutrophils [10] in response to bacterial lipopolysaccharide (LPS) stimulation. The defect of the *NLRP3 *gene results in the hyporesponsiveness or nonresponsiveness of C57BL/6 mouse strains to LPS challenge, showing decreased levels of serum IL-1β and IL-18 and lower mortality [9]. In addition, the expression of the NLRP3 inflammasome mRNAs has been shown to be well associated with the outcomes of patients with sepsis [11].

Growing evidence suggests that genetic variants within the *NLRP3 *gene might be an important determinant of the magnitude of immune inflammatory responses, influencing susceptibility to infectious and noninfectious diseases [12]. Approximately 60 SNPs have been identified within the entire *NLRP3 *gene [13], among which the most often described are rs10754558, rs12065526, rs4612666, rs4925648, rs10925019, rs10925027, rs3806265 and rs35829419. They have been shown to be well associated with susceptibility to HIV infection [14], type 1 diabetes [15], food-induced anaphylaxis [6], rheumatoid arthritis [16], celiac disease [15,17] and Crohn's disease [18], respectively. In spite of the above findings, there are numerous inconsistencies concerning the clinical relevance of these polymorphisms [19-21].

In this study, we hypothesized that the genetic variations in the *NLRP3 *gene might affect the magnitude of immune inflammatory responses following trauma, which subsequently determines the susceptibility to sepsis and MODS in patients with major trauma. To comprehensively assess the association of common genetic variants within the entire *NLRP3 *gene with sepsis susceptibility, we selected a set of tag SNPs (tSNPs) within the entire *NLRP3 *gene and investigated their clinical relevance in relation to the development of sepsis and MODS in patients with major trauma. Two of them were found to be well associated with risk of sepsis and MODS in patients with major trauma.

## Materials and methods

### Study population

A total of 718 unrelated patients with major trauma (577 male and 141 female) were prospectively recruited into this study. All of them are ethnic Han Chinese and live in Chongqing city. All eligible patients were consecutively admitted to the Department of Trauma Surgery in the Daping Hospital and the Chongqing Emergency Medical Center between 1 January 2005 and 1 June 2010. They were enrolled in the study if they met the following entry criteria: (1) age between 18 and 65 years, (2) combined injury patients with expected Injury Severity Score (ISS) > 16 and (3) probability of survival > 48 hours. Patients were not eligible if they had penetrating injuries or preexisting cardiovascular, respiratory, renal, hepatic, hematologic or immunological diseases. ISS were calculated according to the 2005 abbreviated injury scale developed by independent evaluators [22]. All patients requiring surgical intervention received standard surgical care and postoperative ICU treatment. The protocol of the study was approved by the Ethical and Protocol Review Committee of the Third Military Medical University, and informed consent was obtained from the patients or their next of kin. Patient confidentiality was preserved according to the guidelines for studies of human subjects.

### Clinical evaluation

The patients with major trauma were prospectively monitored after admission by physicians who did not know the patients' genotypes. A sepsis diagnosis was made if patients met all of the following criteria: clinical evidence of infection, body temperature > 38.5°C or < 36.5°C and leukocyte count > 10 × 10^9^/L or < 4 × 10^9^/L. Infection was defined as a clinically obvious source or positive bacterial cultures. Pneumonia was diagnosed when a predominant organism was isolated from appropriately obtained sputum cultures in the setting of purulent sputum production and/or a new or changing pulmonary infiltrate on chest X-ray film. Bloodstream infections were diagnosed on the basis of isolation of a predominant organism from blood cultures obtained under sterile conditions. The criteria for urinary tract infections included > 10 white blood cells/high-power field on microscopic examination, isolation of > 10^5 ^organisms/ml urine or > 10^4 ^organisms with symptoms. The criteria for catheter-related infections included isolation of > 15 colony-forming units from catheter tips cultured only in the setting of suspected infection. Wound infection was identified by drainage of purulent material from the wound. Daily physiological and laboratory data were collected during the hospital stays, and clinical events were recorded thereafter until patients' death or hospital discharge. MOD scores were calculated as the sum of the simultaneously obtained individual organ scores on each hospital day [23]. The MOD scores and sepsis diagnoses were determined by individuals who did not know the patients' genotypes.

### Selection of tSNPs and bioinformatics analysis

The full sequence of the human *NLRP3 *gene included 3 kb upstream of the transcription start site, all exons and introns, and 2 kb downstream of the stop codon (37.953 kb total), which was pinpointed to chromosome 1, positions 247576458 to 247614410 [GenBank:114548]. Genetic variation data for the entire *NLRP3 *gene were obtained from the HapMap project for the 45 unrelated Chinese Han in Beijing (CHB) population http://www.ncbi.nlm.nih.gov/projects/SNP/snp_viewTable.cgi?pop_id=4440. In this database, 35 SNPs with a minor allele frequency (MAF) ≥ 5% were identified, and we partitioned them in "bins" inferred according to the *r*^2 ^linkage disequilibrium (LD) statistic (threshold ≥ 0.8) [24]. A maximally informative tSNP was then selected from each bin using TAGster [25]. This algorithm selects a subset of tSNPs that capture all known common genetic variants within the entire gene. To determine the possible functionality of the tSNPs selected from the 5'-flanking region of the *NLRP3 *gene, online software was used to analyze the effect of these SNPs (rs2027432 and rs3738448) on potential transcription factor-binding sites [26].

### Genotyping of tSNPs

Blood specimens were collected in tripotassium ethylenediaminetetraacetic acid sterile tubes from trauma patients immediately after their admission to avoid the effect of blood transfusion. The genomic DNA was isolated from whole blood using the Wizard Genomic DNA Purification Kit (Promega, Madison, WI, USA) according to the manufacturer's protocol. The DNA concentration in all samples was determined by UV spectrophotometry, adjusted to a concentration of 50 μg/ml with sterile distilled water and stored at -80°C. Pyrosequencing was used for genotyping [27]. Genotyping was performed in a blinded fashion without knowledge of the patients' clinical data, and approximately 10% of the samples were genotyped in duplicate to monitor genotyping quality.

### Plasmid construction

The possible effect of rs2027432, which is located in the 5'-flanking region (-1,017 G/A), on the promoter activity was investigated using a reporter gene assay system. In brief, genomic DNA was obtained from patients homozygous for the G allele at position -1,017. The primers used were 5'-TCG ACG CGT CAA GCT ATC CTC CCG CCT CT-3' (forward) and 5'-CCG AAG CTT CAA TCT CCC CCA CTG CTC TC-3' (reverse). *Mlu*I and *Hin*dIII restriction sites (underlined) were introduced through PCRs. After being digested with restriction enzymes *Mlu*I and *Hin*dIII, the PCR products of a 1,760-bp sequence (from about -1,661 to about +99) [28] of the *NLRP3 *gene were inserted directly into a promoterless pGL3-Basic vector (Promega) containing the firefly luciferase gene as a reporter. The resulting construct containing the -1,017 G was then used to generate the construct containing the -1,017 A using the QuikChange Site-Directed Mutagenesis Kit (Agilent Technologies, Inc, Santa Clara, CA, USA) according to the standard protocol. The mutation primers used were 5'-CCA ACT GCT TAT TCT AGC TTC TCT GTG-3' (forward) and 5'-TTC CGA TGA CTA CCA CCT TTC TAC AGA-3' (reverse). All constructs used in this study were restriction-mapped and direct-sequenced (TaKaRa Biotech, Dalian, China) to confirm their authenticity.

### Reporter assay

Human U937 cells were cultured in RPMI 1640 medium (HyClone/Thermo Fisher Scientific, Logan, UT, USA) containing 10% FCS, 3 mM glutamine, penicillin-streptomycin (100 U/ml for each) and 23 mM sodium bicarbonate at 37°C in a humidified 5% CO_2 _air atmosphere. After incubation for 24 hours, the cultured cells were cotransfected with 0.8 μg of the constructed vectors or pGL3-Basic original plasmid and 20 ng of control Renilla luciferase reporter plasmid pRL-CMV using the Lipofectamine 2000 system (Invitrogen/Life Technologies, Carlsbad, CA, USA). At 24 hours posttransfection, the cells were treated with LPS (100 ng/ml) for 24 hours, then the luciferase activity of the transfected cells was measured using the Luciferase Assay System (Promega) following the supplier's protocol on a Luminoskan Ascent microplate luminometer (Thermo Fisher Scientific, Helsinki, Finland). Transfection efficiency was normalized by measuring the luciferase activity of control plasmid pRL-CMV. Luminescence experiments were performed in triplicate with each transfection. Three independent transfections were performed for each constructed vector. The results are expressed as fold increases in relative luciferase activity of the NLRP3 promoter construct vectors compared with the relative luciferase activity of pGL3-Basic.

### *Ex vivo *LPS stimulation of whole blood

A human whole-blood assay was performed as described previously [29]. In brief, aliquots of whole blood collected from the trauma patients immediately after admission were mixed at 1:1 dilution with RPMI 1640 culture medium and incubated with 100 ng/ml LPS (*Escherichia coli *O26:B6; Difco Laboratories/BD Microbiology Systems, Detroit, MI, USA) in a sample mixer at 37°C for 4 hours. The supernatants were carefully collected after centrifugation and stored at -80°C for assays of IL-1β production, which was determined by ELISA according to the manufacturer's instructions (R&D Systems, Minneapolis, MN, USA), the theoretical sensitivity (as stated by the manufacturer) of the immunoassay was 2 pg/ml.

### Statistical analysis

Sample size was calculated using the online Power and Sample Size software program [30]. The desired power of our study was set at 80% with a significance level of 0.05 calculated using a two-sided test. We chose the log-additive inheritance model, which is the most suitable one for polygenic diseases. On the basis of our calculations using the Power and Sample Size software program, our sample (*N *= 718) was considered adequate to study the tSNPs of the *NLRP3 *gene.

Allele frequencies for each tSNP were determined by gene counting. The genotype distribution of each tSNP was analyzed for deviations from the Hardy-Weinberg equilibrium using χ^2 ^analyses. The extent of pairwise LD (*r*^2^-value) between polymorphisms was determined using Haploview version 4.1 software. The association of the *NLRP3 *gene polymorphisms with plasma IL-1β levels was determined using one-way analysis of variance. The association between polymorphisms and MOD scores was assessed using analysis of covariance testing with age, sex ratio and ISS to adjust for possible confounding effects. Three genetic models were used (allele-dose, dominant, and recessive). The association of genotypes with sepsis morbidity rate was determined by χ^2 ^analysis. ORs with 95% confidence intervals were calculated by using multivariate logistic regression models to estimate the relative risk of sepsis. All *P*-values were two-sided, and *P *< 0.05 after the Bonferroni correction for multiple testing was defined as statistically significant. All statistical analysis was carried out using the SPSS version 11.5 statistical software package (SPSS, Inc, Chicago, IL, USA).

## Results

### Construction of bins and selection of tSNPs

**Figure 1:**
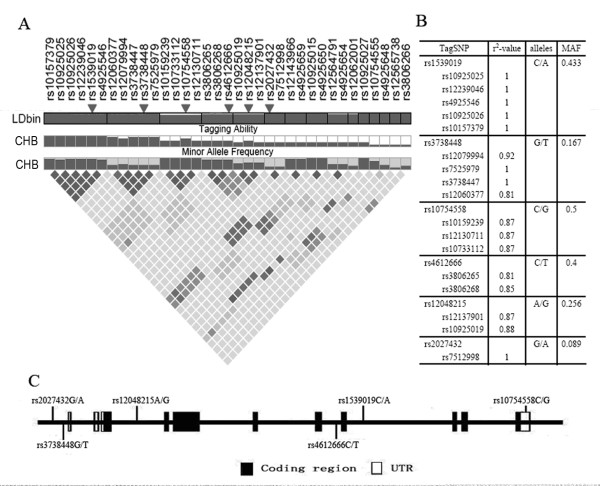
**Overview of selected tag SNPs within the entire *NLRP3 *gene**. **(A) **The pairwise analysis of linkage disequilibrium (LD), based on *r*^2^, among the 35 SNPs with a minor allele frequency (MAF) ≥ 5% within the *NLRP3 *gene and the 5-kb up- and downstream regions. The selected six tag SNPs (tSNPs) are indicated by trigones. A LD plot of the 35 SNPs in the 37.953-kb region is displayed by using an *r*^2 ^black-and-white color scheme. Black represents very high LD correlation between SNPs (*r*^2 ^= 0.8 to 1), and white indicates the absence of correlation between SNPs (*r*^2 ^= 0 to 0.2). **(B) **The six tSNPs and SNPs that are indirectly measured by tSNPs are listed with corresponding *r*^2 ^values. Major and minor alleles of the selected tSNPs are given with their frequencies, based on the HapMap data for the Chinese Han Beijing (CHB) population. **(C) **The exon-intron structure of the *NLRP3 *gene and the locations of the tSNPs are shown. rs2027432 (-1,017 G/A) and rs3738448 (-23 G/T) map to within the 5'-flanking region. rs12048215 (5,134 A/G), rs4612666 (19,613 C/T) and rs1539019 (20,844 C/A) are located in introns 3, 7 and 8, and rs10754558 (32,579 C/G) is located in the 3'-untranslated region (3'-UTR).

A total of 35 SNPs with a minor allele frequency ≥ 0.05 were shown within and around the *NLRP3 *gene from the HapMap database for the CHB population. Five bins were constructed among 23 SNPs based on the *r*^2 ^LD statistic (threshold ≥ 0.8) (Figure 1A). One tSNP was selected from each bin for genotyping (Figure 1B). rs2027432, though it did not form any bin with other SNPs, was located in the 5'-flanking region (-1,017 G/A) and selected because of its potential effect on the binding of transcription factor MyoD. Therefore, six SNPs (rs2027432, rs3738448, rs12048215, rs4612666, rs1539019 and rs10754558) were selected from among this study cohort. They were located in the 5'-flanking region (rs2027432 and rs3738448), intron 3 (rs12048215), intron 7 (rs4612666), intron 8 (rs1539019) and 3'-UTR (rs10754558), respectively (Figure 1C).

### Allele frequencies and genotype distribution of the *NLRP3 *gene polymorphisms among trauma patients

The genotyping success rates of the six tSNPs by pyrosequencing ranged from 98.2% to 100% in our study cohort. The MAFs among the 718 trauma patients were 5.5% (rs2027432), 19.2% (rs3738448), 26.0% (rs12048215), 46.1% (rs4612666), 40.7% (rs1539019) and 40.8% (rs10754558), respectively, which were quite similar to those observed in the 45 unrelated CHB cohort in the HapMap database. The genotype distribution of all six tSNPs was in agreement with the Hardy-Weinberg equilibrium (*P *> 0.05) (Table 1), indicating that the allele and genotype frequencies of these tSNPs in the population remain constant. That is to say, they are in equilibrium from generation to generation.

**Table 1:** Distribution of the six tag SNPs of the *NLRP3 *gene among trauma patients

		MAF (%)		Genotypes, *n *(%)			
			
SNPs	Number of patients	Patients	Database*	Wild type	Heterozygous	Variant	HWE test
rs2027432	718	5.5	8.9	642 (89.4)	73 (10.2)	3 (0.4)	0.73
rs3738448	705	19.2	16.7	454 (64.4)	231 (32.8)	20 (2.8)	0.18
rs12048215	718	26.0	25.6	398 (55.4)	267 (37.2)	53 (7.4)	0.42
rs4612666	711	46.1	40	193 (27.1)	380 (53.5)	138 (19.4)	0.06
rs1539019	718	40.7	43.3	251 (35.0)	349 (48.6)	118 (16.4)	0.93
rs10754558	714	40.8	50	246 (34.5)	354 (49.6)	114 (15.9)	0.54

*Data are derived from the HapMap database for Chinese Han in Beijing (*n *= 45). HWE = Hardy-Weinberg equilibrium; MAF = minor allele frequency.

### Clinical association of *NLRP3 *gene polymorphisms with development of sepsis and MODS in trauma patients

The patient cohort comprised a total of 718 consecutive Han Chinese patients, 577 male and 144 female, with mean ± SD age of 40.6 ± 13.8 years and a mean ± SD ISS of 22.5 ± 10.0. The demographic and baseline characteristics and the clinical data from 718 severe trauma patients are summarized in Table 2. All patients survived ≥ 48 hours after admission. Two hundred eighty-seven patients (40.0%) developed sepsis. Pathogens were identified as causative microorganisms in the blood cultures of 124 septic patients (43.2%). The common pathogens identified in this study cohort were *Staphylococcus aureus*, coagulase-negative staphylococci, *Klebsiella pneumoniae*, *Acinetobacter baumanii*, *Pseudomonas aeruginosa*, *Escherichia coli*, *Enterococcus *sp. and *Enterobacter cloacae*. Gram-negative infections accounted for 17.1%, Gram-positive infections accounted for 6.3% and mixed infections accounted for 11.1%, respectively. The median time point for sepsis occurrence in the whole study cohort was 7 days (IQR 5.0 to 8.0 days). Organ dysfunction occurred in 299 patients (41.6%), from among whom 84 (28.1%) had two or more organ dysfunctions. Among the patients with MODS, those with sepsis accounted for 70.2%. Among the patients with sepsis, the median time point for MODS occurrence was 8 days (IQR = 6.5 to 10.5 days). With respect to the patients without sepsis, the median time point for MODS occurrence was 5 days (IQR = 4 to 8 days).

**Table 2:** Overall clinical characteristics of patients with major trauma

Clinical characteristics	Patient data (*N *= 718)
Mean age ± SD (years)	40.6 ± 13.8
Age range (years)	18 to 65
Males/females, *n*	577/141
Mean ISS ± SD	22.5 ± 10.0
≥ 16 to < 25, *n*	413
≥ 25, *n*	305
Injured body regions, *n*	
Head	385
Thorax	424
Abdomen	249
Extremities	431
Number of regions injured, *n*	
Two	306
Three	177
All four	37
Organ dysfunction, *n *(%)	299 (41.6%)
One, *n*	215
Two, *n*	57
Three or above, *n*	27
Sepsis, *n *(%)	287 (40.0%)
Source of infection (%)	
Respiratory tract infection	42.5
Primary bloodstream infection	20.2
Urinary tract infection	18.3
Catheter-associated infection	9.8
Wound infection	7.1
Others*	2.1
Pathogens (positive blood cultures) (%)	
Gram-negative	17.1
Gram-positive	6.3
Fungi	8.7
Mixed Gram-negative and Gram-positive	11.1
Negative blood cultures	56.8

*Other sites of infection included soft-tissue infection, bone infection and ear infection. ISS = Injury Severity Score.

As shown in Table 3, there were no significant differences in age, sex ratio or ISS among patients stratified according to the different genotypes of each tSNP. The rs2027432 polymorphism, although it was found in only three patients with variant homozygotes in this study cohort, was significantly associated with higher risk of MODS. It was associated with significantly higher MOD scores in patients with the A variant allele than in patients with the G allele (*P *= 0.024 in case of recessive effect). The sepsis morbidity rate was also much higher in patients with the A variant allele, with 66.7% in AA and 43.8% in GA vs 39.3% in GG, although there was no statistically significant difference between the different genotypes. In addition, the rs12048215 polymorphism was significantly associated with a lower sepsis morbidity rate, showing 26.4% in GG and 36.6% in AG vs 44% in AA (*P *= 0.012 in case of dominant effect and *P *= 0.038 in case of recessive effect). Data from multiple logistic regression analyses further indicated that the patients with the rs12048215 polymorphism had a lower risk for developing sepsis (OR = 0.692, 95% CI = 0.535 to 0.896; *P *= 0.005) after adjusting for possible confounders, including age, sex ratio and ISS. However, this polymorphism was not associated with MOD scores. There were no significant associations with sepsis morbidity rate and MOD scores for the other four SNPs (rs3738448, rs4612666, rs1539019 and rs10754558). Concerning the combined effect between the rs2027432 and rs12048215 polymorphisms, no significant differences were observed in sepsis morbidity rate (46.1% vs 41.1%; *P *= 0.409) and MOD scores (4.3 ± 0.4 vs. 4.2 ± 0.1; *P *= 0.739) between patients with combined and single-variant genotypes (AA-AA + GA-AA + GA-AG vs GG-AA + GG-AG).

**Table 3:** Clinical relevance of the *NLRP3 *gene polymorphisms in patients with major trauma

Polymorphic sites	Genotypes	Number of patients	Mean age ± SD (years)	Sex (M/F)	Mean ISS ± SD	Sepsis, *n *(%)	Mean MOD score ± SEM
	GG	642	40.6 ± 13.8	513/129	22.5 ± 10.0	252 (39.3)	4.2 ± 0.1
rs2027432	GA	73	40.9 ± 12.9	60/13	22.4 ± 9.8	32 (43.8)	4.1 ± 0.4
	AA	3	49.0 ± 26.1	3/0	21.7 ± 10.3	2 (66.7)	8.7 ± 1.2
*P*-value							a1
	GG	454	40.5 ± 13.5	369/85	22.2 ± 9.9	173 (38.1)	4.0 ± 0.2
rs3738448	GT	231	40.9 ± 14.4	176/55	22.9 ± 10.2	95 (41.1)	4.3 ± 0.2
	TT	20	40.7 ± 13.3	18/2	22.4 ± 10.0	11 (55)	4.4 ± 0.8
	AA	398	40.2 ± 13.8	320/78	22.3 ± 9.8	175 (44.0)	4.3 ± 0.2
rs12048215	AG	267	40.5 ± 13.6	217/50	22.9 ± 10.5	97 (36.3)	4.1 ± 0.2
	GG	53	44.7 ± 14.1	39/14	21.4 ± 8.0	14 (26.4)	3.7 ± 0.1
*P*-value						a2, b1	
	CC	193	39.6 ± 13.8	165/28	21.7 ± 9.3	76 (39.4)	4.0 ± 0.2
rs4612666	CT	380	40.6 ± 13.4	295/85	22.9 ± 10.6	156 (41.1)	4.2 ± 0.2
	TT	138	42.4 ± 14.7	109/29	22.4 ± 9.2	49 (35.5)	4.0 ± 0.3
	CC	251	40.1 ± 14.1	195/56	22.4 ± 9.4	111 (44.2)	4.2 ± 0.2
rs1539019	CA	349	40.4 ± 13.7	283/66	23.1 ± 10.1	129 (37.0)	4.3 ± 0.2
	AA	118	40.0 ± 13.0	98/20	20.9 ± 10.5	46 (39.0)	3.9 ± 0.3
	CC	246	40.5 ± 14.9	186/60	21.6 ± 9.1	99 (40.2)	3.9 ± 0.2
rs10754558	CG	354	40.6 ± 13.2	291/62	23.0 ± 10.3	134 (37.9)	4.4 ± 0.2
	GG	114	40.3 ± 12.5	96/18	22.6 ± 10.7	51 (44.7)	3.9 ± 0.3

ISS = Injury Severity Score; MOD score = Multiple Organ Dysfunction score. a = recessive effect (variant homozygotes versus heterozygotes + wild-type homozygotes); b = dominant effect (variant homozygotes + heterozygotes versus wild-type homozygotes) as analyzed by one-way analysis of variance. a1, *P *= 0.024; a2, *P *= 0.038; b1, *P *= 0.012.

### Association of rs2027432 and rs12048215 polymorphisms with IL-1β production

**Figure 2:**
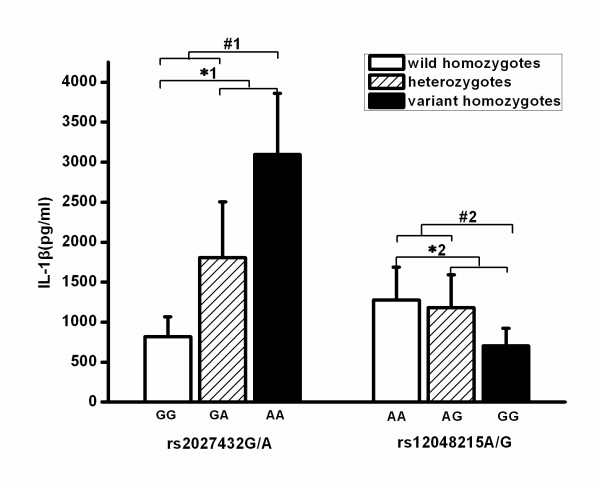
**Effect of the rs2027432 and rs12048215 polymorphisms of the *NLRP3 *gene on IL-1β production**. The whole-blood samples collected from trauma patients immediately after admission were mixed at a 1:1 ratio (vol/vol) with RPMI 1640 culture medium and incubated with 100 ng/ml bacterial lipopolysaccharide at 37°C for 4 hours. The levels of IL-1β in the supernatants were assayed by performing a sandwich ELISA. The data are presented as means ± SD. One-way analysis of covariance (ANCOVA) was used to assess statistical significance. *Dominant effect (variant homozygotes + heterozygotes vs wild homozygotes) assessed by ANCOVA: *^1^*P *= 0.003, *^2^*P *= 0.018. ^#^Recessive effect (variant homozygotes vs heterozygotes + wild-type homozygotes) assessed by ANCOVA: ^#1^*P *< 0.0001, ^#2^*P *= 0.00016.

NLRP3 is a key molecule for the maturation and secretion of IL-1β [31]. Therefore, we hypothesized that the rs2027432 and rs12048215 polymorphisms might be associated with IL-1β production. Peripheral blood was taken from patients immediately after admission in an attempt to avoid the potential effects of nongenetic factors, and we stimulated the samples with LPS for IL-1β production. As shown in Figure 2, these two polymorphisms were significantly associated with IL-1β production. The rs2027432 polymorphism was significantly associated with higher IL-1β production, with IL-1β levels significantly higher in patients with the variant A allele than in those with the wild-type G allele (*P *= 0.003 for dominant effect and *P *< 0.0001 for recessive effect). The rs12048215 polymorphism was significantly associated with lower IL-1β production (*P *= 0.018 for dominant effect and *P *= 0.00016 for recessive effect). Data from linear regression analysis indicated that the association of these two polymorphisms with IL-1β production was significantly in allele-dose dependent (*P *< 0.0001 for rs2027432 and *P *= 0.001 for rs12048215). With respect to their combined effect on IL-1β production, no significant difference was observed between patients with combined and single-variant genotypes (AA-AA + GA-AA + GA-AG vs GG-AA + GG-AG) (1,483.2 ± 889.2 pg/ml vs 1,238.2 ± 363.9 pg/ml; *P *= 0.343).

### Effect of the rs2027432 polymorphism on promoter activity

**Figure 3:**
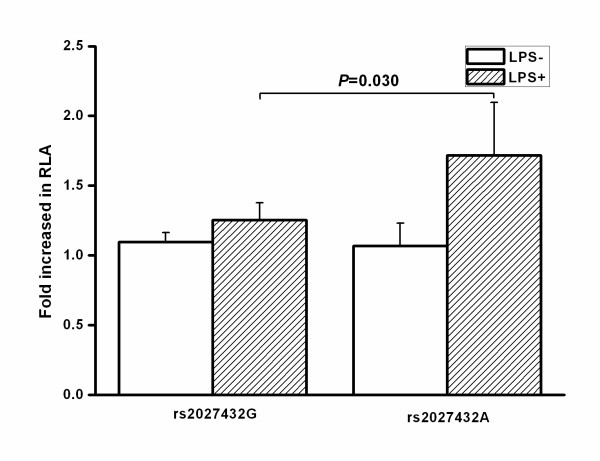
**Effect of the rs2027432 polymorphism on the transcription activity of the NLRP3 promoter**. Relative luciferase activity (RLA) was measured in human U937 cells transfected with rs2027432G or rs2027432A plasmid constructs as described in Methods. Luciferase activity was normalized for transfection efficiency by using a control plasmid, pRL-CMV. The results are expressed as fold increases in RLA of the NLRP3 promoter construct vector as compared with pGL3-Basic (means ± SD). LPS = lipopolysaccharide.

In view of the location of the rs2027432 polymorphism in the 5'-flanking region of the *NLRP3 *gene, we further hypothesized that the G → A variation of the rs2027432 polymorphism might affect the promoter activities of the *NLRP3 *gene. Figure 3 shows that the fold increase of RLA was significantly higher in cells transfected with the variant A allele than in those transfected with wild-type G allele in the presence of LPS stimulation (1.72 vs 1.25; *P *= 0.03), whereas there was no statistical significance between the two groups in the absence of LPS stimulation (1.07 vs 1.09; *P *= 0.807).

## Discussion

In the present study, to the best of our knowledge, we are the first investigators to identify the potential clinical relevance of the rs2027432, rs3738448, rs12048215, rs4612666, rs1539019 and rs10754558 polymorphisms, which are identified as tSNPs of the entire *NLRP3 *gene in the Han Chinese population. tSNPs are a subset of all variants within a chromosome region within a disease study. The genetic effect of SNPs that are not genotyped in the study can be detected through LD with a tSNP [32]. In this case, the six tSNPs selected in the current study theoretically reflect the biological significance of all the genetic variations across the entire *NLRP3 *gene because of their strong LD with other unassayed variants.

A case-control study is a common and convenient association study design for finding a genetic basis of disease. However, a major limitation of this approach is the potential for population stratification when inappropriate patient-control matching occurs, such as using healthy blood donors as the control group in the study. To avoid confounding associations, we selected only trauma patients and followed them prospectively to determine whether those who had genetic variants had a lesser or higher risk of posttraumatic MODS and sepsis. In addition, we limited the patients recruited into this study cohort to Han Chinese to avoid an artefact due to population admixture. In the context of a biologically relevant phenotype and a racially uniform population, this might maximize the likelihood of finding a meaningful genetic association. As shown by our clinical association study, among the six tSNPs selected in our study cohort, only two of them (rs2027432 and rs12048215) had clinical relevance in relation to the development of sepsis and MODS in trauma patients. The rs2027432 polymorphism reveals higher risk with the development of sepsis and MODS, in which there is a significant association with MODS. The lack of a statistically significant association with sepsis might be due to the very small number of patients with the AA genotype (*n *= 3), which is not quite enough for χ^2 ^analysis. For the rs12048215 polymorphism, the number of patients appears to be big enough for each genotype group (*n *= 398 for AA, *n *= 267 for AG and *n *= 53 for GG). It has a significant association with lower risk of sepsis and only a trend association with MODS. It suggests that the rs2027432 polymorphism has stronger clinical relevance than rs12048215. In addition, rs2027432 and rs12048215 did not have a combined effect in relation to risk of development of sepsis and MODS, with no significant differences in sepsis morbidity rate and MOD scores observed between trauma patients with combined and single-variant genotypes (AA-AA + GA-AA + GA-AG vs GG-AA + GG-AG). The rs1539019, rs4612666C and rs10754558 polymorphisms, although well associated with heart disease [33], food-induced anaphylaxis and aspirin-induced asthma [6], HIV-1 infection [14] and type 1 diabetes [15], respectively, showed negative results in our study cohort. Different ethnic populations and diseases might account for these opposite results.

Given the clinical relevance of both the rs2027432 and rs12048215 polymorphisms, we further hypothesized that both genetic variations might be associated with IL-1β production in patients with major trauma. The peripheral blood was taken immediately after admission in an attempt to avoid the potential effects of infection and fluid resuscitation. Our results reveal that the two polymorphisms could affect the capacity of peripheral leukocytes to produce IL-1β, showing significant association with higher (rs2027432) and lower (rs12048215) IL-1β production, respectively. But these two polymorphisms do not have a combined effect on IL-1β production. This is in accordance with their clinical relevance. rs12048215 is located in the intron of the *NLRP3 *gene. Unfortunately, it is unclear how a genetic variant in an intron affects the function of the gene. The effect of the rs12048215 on IL-1β production might be due to other, unidentified causal SNPs, which is in close LD with the rs12048215. The rs2027432 polymorphism is located in the 5'-flanking region of the *NLRP3 *gene, causing G-to-A variation at position -1,017. To further determine whether the association of the rs2027432 polymorphism with IL-1β production is due to the direct effect of this polymorphism, we further investigated the effect of the rs2027432 polymorphism on NLRP3 promoter activity using a reporter gene assay system. Our results indicate that the G → A variation at position -1,017 could significantly enhance the transcriptional activity of the NLRP3 promoter, leading to increased expression of the *NLRP3 *gene. Results from bioinformatics analysis further indicate that the mechanism of the rs2027432 polymorphism's affecting promoter activity might be due to its effect on the binding of transcription factor MyoD, which might be further confirmed by a electrophoretic mobility shift assay. NLRP3 has been recognized as a central molecule in the maturation and secretion of IL-1β [31]. The rs2027432 polymorphism might enhance IL-1β production through upregulation of the expression of the *NLRP3 *gene.

## Conclusions

In the present study, we investigated the clinical relevance of the genetic variations within the entire *NLRP3 *gene by means of constructing haplotype bins in patients with major trauma. We have demonstrated that the rs2027432 and rs12048215 polymorphisms affect IL-1β production and might be used to estimate risk for sepsis and MODS in trauma patients. Further studies, both clinical and experimental, are therefore needed to confirm the significance of these findings and to investigate their synergistic effect with other genetic polymorphisms in relation to the development of sepsis in and the outcomes of trauma patients.

## Key messages

• The rs2027432A and rs12048215A alleles were significantly associated with higher IL-1β production in response to *ex vivo *LPS stimulation.

• The rs2027432 and rs12048215 polymorphisms were closely associated with the development of sepsis and MODS.

• There are no marked synergistic effects among the rs2027432 and rs12048215 polymorphisms in relation to the development of sepsis and MODS in trauma patients.

## Abbreviations

ELISA: enzyme-linked immunosorbent assay; FCS: fetal calf serum; IL: interleukin; LPS: lipopolysaccharide; MODS: multiple organ dysfunction syndrome; NLRP3: NLR family, pyrin domain containing 3; PCR: polymerase chain reaction; SNP: single-nucleotide polymorphism; UTR: untranslated region.

## Competing interests

The authors declare that they have no competing interests.

## Authors' contributions

AQZ and LZ were the main researchers in this study, and both contributed to writing the manuscript. WG, LYZ, JZ, DPJ, DYD, PH, CY and JY were involved in collecting the blood samples and clinical data. HYW did the technical work. JXJ planned the study, wrote the protocol, was involved in the genetic and clinical aspects of data analyses, and revised the manuscript. All authors read and approved the final manuscript for publication.

## References

[B1] Wang Z Jiang J An overview of research advances in road traffic trauma in China Traffic Inj Prev 2003 4 9 16 10.1080/15389580309860 14522656

[B2] Neunaber C Zeckey C Andruszkow H Frink M Mommsen P Krettek C Hildebrand F Immunomodulation in polytrauma and polymicrobial sepsis: where do we stand? Recent Pat Inflamm Allergy Drug Discov 2011 5 17 25 10.2174/187221311794474892 21158733

[B3] Gu W Shan YA Zhou J Jiang DP Zhang L Du DY Wang ZG Jiang JX Functional significance of gene polymorphisms in the promoter of myeloid differentiation-2 Ann Surg 2007 246 151 158 10.1097/01.sla.0000262788.67171.3f 17592304 PMC1899213

[B4] Wen AQ Wang J Feng K Zhu PF Wang ZG Jiang JX Effects of haplotypes in the interleukin 1β promoter on lipopolysaccharide-induced interleukin 1β expression Shock 2006 26 25 30 16783194 10.1097/01.shk.0000223125.56888.c7

[B5] Martinon F Burns K Tschopp J The inflammasome: a molecular platform triggering activation of inflammatory caspases and processing of proIL-β Mol Cell 2002 10 417 426 10.1016/S1097-2765(02)00599-3 12191486

[B6] Hitomi Y Ebisawa M Tomikawa M Imai T Komata T Hirota T Harada M Sakashita M Suzuki Y Shimojo N Kohno Y Fujita K Miyatake A Doi S Enomoto T Taniguchi M Higashi N Nakamura Y Tamari M Associations of functional *NLRP3 *polymorphisms with susceptibility to food-induced anaphylaxis and aspirin-induced asthma J Allergy Clin Immunol 2009 124 779 785.e6 10.1016/j.jaci.2009.07.044 19767079

[B7] Tschopp J Schroder K NLRP3 inflammasome activation: the convergence of multiple signalling pathways on ROS production? Nat Rev Immunol 2010 10 210 215 10.1038/nri2725 20168318

[B8] Fritz JH Ferrero RL Philpott DJ Girardin SE Nod-like proteins in immunity, inflammation and disease Nat Immunol 2006 7 1250 1257 10.1038/ni1412 17110941

[B9] Sutterwala FS Ogura Y Szczepanik M Lara-Tejero M Lichtenberger GS Grant EP Bertin J Coyle AJ Galán JE Askenase PW Flavell RA Critical role for NALP3/CIAS1/cryopyrin in innate and adaptive immunity through its regulation of caspase-1 Immunity 2006 24 317 327 10.1016/j.immuni.2006.02.004 16546100

[B10] Kummer JA Broekhuizen R Everett H Agostini L Kuijk L Martinon F van Bruggen R Tschopp J Inflammasome components NALP 1 and 3 show distinct but separate expression profiles in human tissues suggesting a site-specific role in the inflammatory response J Histochem Cytochem 2007 55 443 452 10.1369/jhc.6A7101.2006 17164409

[B11] Fahy RJ Exline MC Gavrilin MA Bhatt NY Besecker BY Sarkar A Hollyfield JL Duncan MD Nagaraja HN Knatz NL Hall M Wewers MD Inflammasome mRNA expression in human monocytes during early septic shock Am J Respir Crit Care Med 2008 177 983 988 10.1164/rccm.200703-418OC 18263805 PMC2361424

[B12] Verma D Lerm M Blomgran Julinder R Eriksson P Söderkvist P Särndahl E Gene polymorphisms in the NALP3 inflammasome are associated with interleukin-1 production and severe inflammation: relation to common inflammatory diseases? Arthritis Rheum 2008 58 888 894 10.1002/art.23286 18311798

[B13] International HapMap Project: HapMap Database http://snp.cshl.org/cgi-perl/gbrowse/hapmap27_B36/

[B14] Pontillo A Brandão LA Guimarães RL Segat L Athanasakis E Crovella S A 3'UTR SNP in NLRP3 gene is associated with susceptibility to HIV-1 infection J Acquir Immune Defic Syndr 2010 54 236 240 10.1097/QAI.0b013e3181dd17d4 20502346

[B15] Pontillo A Brandão LA Guimarães RL Segat L Araujo J Crovella S Two SNPs in *NLRP3 *gene are involved in the predisposition to type-1 diabetes and celiac disease in a pediatric population from northeast Brazil Autoimmunity 2010 43 583 589 10.3109/08916930903540432 20370570

[B16] Kastbom A Verma D Eriksson P Skogh T Wingren G Söderkvist P Genetic variation in proteins of the cryopyrin inflammasome influences susceptibility and severity of rheumatoid arthritis (the Swedish TIRA project) Rheumatology (Oxford) 2008 47 415 417 18263599 10.1093/rheumatology/kem372

[B17] Pontillo A Vendramin A Catamo E Fabris A Crovella S The missense variation Q705K in CIAS1/NALP3/NLRP3 gene and an NLRP1 haplotype are associated with celiac disease Am J Gastroenterol 2011 106 539 544 10.1038/ajg.2010.474 21245836

[B18] Schoultz I Verma D Halfvarsson J Törkvist L Fredrikson M Sjöqvist U Lördal M Tysk C Lerm M Söderkvist P Söderholm JD Combined polymorphisms in genes encoding the inflammasome components NALP3 and CARD8 confer susceptibility to Crohn's disease in Swedish men Am J Gastroenterol 2009 104 1180 1188 10.1038/ajg.2009.29 19319132

[B19] Villani AC Lemire M Fortin G Louis E Silverberg MS Collette C Baba N Libioulle C Belaiche J Bitton A Gaudet D Cohen A Langelier D Fortin PR Wither JE Sarfati M Rutgeerts P Rioux JD Vermeire S Hudson TJ Franchimont D Common variants in the *NLRP3 *region contribute to Crohn's disease susceptibility Nat Genet 2009 41 71 76 10.1038/ng.285 19098911 PMC2728932

[B20] Lewis GJ Massey DC Zhang H Bredin F Tremelling M Lee JC Berzuini C Parkes M Genetic association between NLRP3 variants and Crohn's disease does not replicate in a large UK panel Inflamm Bowel Dis 2011 17 1387 1391 10.1002/ibd.21499 21560198

[B21] Wang W Stassen FR Surcel HM Ohman H Tiitinen A Paavonen J de Vries HJ Heijmans R Pleijster J Morré SA Ouburg S Analyses of polymorphisms in the inflammasome-associated NLRP3 and miRNA-146A genes in the susceptibility to and tubal pathology of *Chlamydia trachomatis *infection Drugs Today (Barc) 2009 45 Suppl B 95 103 20011700

[B22] Association for the Advancement of Automotive Medicine (AAAM) The Abbreviated Injury Scale 2005 Barrington, IL: AAAM

[B23] Marshall JC Cook DJ Christou NV Bernard GR Sprung CL Sibbald WJ Multiple Organ Dysfunction Score: a reliable descriptor of a complex clinical outcome Crit Care Med 1995 23 1638 1652 10.1097/00003246-199510000-00007 7587228

[B24] Carlson CS Eberle MA Rieder MJ Yi Q Kruglyak L Nickerson DA Selecting a maximally informative set of single-nucleotide polymorphisms for association analyses using linkage disequilibrium Am J Hum Genet 2004 74 106 120 10.1086/381000 14681826 PMC1181897

[B25] Xu Z Kaplan NL Taylor JA TAGster: efficient selection of LD tag SNPs in single or multiple populations Bioinformatics 2007 23 3254 3255 10.1093/bioinformatics/btm426 17827206 PMC2782964

[B26] MOTIF: Searching Sequence Protein Motifs http://www.genome.jp/tools/motif/

[B27] Chen KH Gu W Zeng L Jiang DP Zhang LY Zhou J Du DY Hu P Liu Q Huang SN Jiang JX Identification of haplotype tag SNPs within the entire *TLR2 *gene and their clinical relevance in patients with major trauma Shock 2011 35 35 41 10.1097/SHK.0b013e3181eb45b3 20577149

[B28] Anderson JP Mueller JL Misaghi A Anderson S Sivagnanam M Kolodner RD Hoffman HM Initial description of the human NLRP3 promoter Genes Immun 2008 9 721 726 10.1038/gene.2008.66 18719602 PMC4477692

[B29] Chen K Wang YT Gu W Zeng L Jiang DP Du DY Hu P Duan ZX Liu Q Huang SN Jiang JX Functional significance of the Toll-like receptor 4 promoter gene polymorphisms in the Chinese Han population Crit Care Med 2010 38 1292 1299 20228685 10.1097/CCM.0b013e3181d8ad12

[B30] Power and Sample Size Calculation http://biostat.mc.vanderbilt.edu/twiki/bin/%20view/Main/PowerSampleSize

[B31] Weber A Wasiliew P Kracht M Interleukin-1β (IL-1β) processing pathway Sci Signal 2010 3 cm2 10.1126/scisignal.3105cm2 20086236

[B32] Barrett JC Fry B Maller J Daly MJ Haploview: analysis and visualization of LD and haplotype maps Bioinformatics 2005 21 263 265 10.1093/bioinformatics/bth457 15297300

[B33] Dehghan A Yang Q Peters A Basu S Bis JC Rudnicka AR Kavousi M Chen MH Baumert J Lowe GD McKnight B Tang W de Maat M Larson MG Eyhermendy S McArdle WL Lumley T Pankow JS Hofman A Massaro JM Rivadeneira F Kolz M Taylor KD van Duijn CM Kathiresan S Illig T Aulchenko YS Volcik KA Johnson AD Uitterlinden AG Association of novel genetic loci with circulating fibrinogen levels: a genome-wide association study in 6 population-based cohorts Circ Cardiovasc Genet 2009 2 125 133 10.1161/CIRCGENETICS.108.825224 20031576 PMC2764985

